# The Efficacy of Acupuncture for the Treatment of Cervical Vertigo: A Systematic Review and Meta-Analysis

**DOI:** 10.1155/2017/7597363

**Published:** 2017-05-09

**Authors:** Zhuanzhuan Hou, Shibing Xu, Qinglin Li, Libing Cai, Weigang Wu, Huida Yu, Huade Chen

**Affiliations:** ^1^The Third People's Hospital of Xiaoshan, Hangzhou, Zhejiang 311200, China; ^2^Zhejiang Chinese Medical University, Hangzhou, Zhejiang 310053, China; ^3^Zhejiang Cancer Hospital, Hangzhou, Zhejiang 310022, China

## Abstract

**Objective:**

This study aims to evaluate the efficacy and safety of acupuncture for the treatment of cervical vertigo (CV).

**Methods:**

Randomized controlled trials (RCTs) regarding effectiveness of acupuncture for treating CV were searched in 7 comprehensive databases prior to April 2016. The data analysis was performed by using RevMan version 5.3.

**Results:**

A total of 10 studies with 914 participants were included. Results showed that acupuncture was more effective than conventional medicine therapy (CMT) in effectiveness, improvement rate of vertigo and headache, and increased average blood flow velocity of vertebral-basilar artery. In the subgroup analysis, the results did not change in different acupuncture methods and drug categories substantially. Sensitivity analysis demonstrated that the results of this meta-analysis were stable. Meanwhile, the long-term safety of acupuncture for CV still remains uncertain. GRADE analysis indicated that the quality of evidence for all outcomes was from very low to low which limited the value of the meta-analysis.

**Conclusion:**

Based on the systematic review, acupuncture appeared to be a promising therapeutic approach for CV based on low or very low quality of evidence. However, large-scale and high-quality trials are required to provide stronger evidence for the conclusion.

## 1. Introduction

In the western medicine, cervical vertigo (CV) is a clinical syndrome caused by hyperostosis of cervical vertebra and degeneration of cervical intervertebral disc. Ryan and Cope put forward the concept, that is, cervical vertigo, in 1955 [1]. CV often has clinical manifestations of narrowing and insufficient blood supply of vertebral artery, such as dizziness, blurred vision, headache, nausea, vomiting, and even fainting [2]. These symptoms can be induced and aggravated by turning head and bending neck laterally to a certain position. With the change of the people's lifestyle, the incidence of CV is rising and tends to be younger. The study found that the adult incidence of the disease is 10% [3]. This disease can break out repeatedly, seriously develop for stroke [4], and bring bigger challenges to clinical treatment. Western medicine thinks [5] that hyperplasia and hypertrophy of the cervical vertebra, the movement of the vertebral body, and indirect compression from vertebral artery and the sympathetic nerve are the external factors of CV, and vertebral atherosclerosis and decreased vascular elasticity caused by abnormal hemodynamics are its pathological basis [6]. In the recent years, western medicine treatment including drug and surgery therapy had difficulty in obtaining satisfactory effect [7].

According to traditional Chinese medicine (TCM), CA belongs to the category of “vertigo.” The disease's region is in the brain and quality is deficiency in origin and enrichment in symptom. Many doctors in generations analyzed disease's pathogenesis from different views; they thought that wind, fire, phlegm, blood stasis, and deficiency played an important role in CV. Pathogen invades Du Meridian and then goes upwards to the brain for causing vertigo. Acupuncture is widely used in clinical practice in China and many western countries [8]. Acupuncture has been used for relief of these illnesses such as pain, dizziness, and vertigo in TCM over a thousand years [9, 10]. Recent studies [11–13] have suggested that acupuncture may have promising therapeutic effectiveness for CV. It was reported that acupuncture has positive effect in releasing cervical surrounding tissue, regulating the flow of Qi and blood, nourishing the brain in order to improve cerebral blood supply [14]; for example, Fengchi (GB 20), Baihui (GV 20), and Lieque (LU 7) were thought to accelerate velocity of blood flow and improve blood supply to the vertebral arteries [15, 16]. Min et al. drew a conclusion that the increased blood perfusion induced by acupuncture stimulation might be relevant to the suppression of the sympathetic nerve activity and the local action of vasodilation factors such as substance P and CGRP [17, 18]. These cases made us wonder whether or not acupuncture has some real benefits to the sufferers. Some systematic reviews [19, 20] had proven limited evidence that acupuncture has effectiveness for CV. We carried out a comprehensive and quantitative evaluation analysis to assess its efficacy and safety in the clinical treatment of this condition.

## 2. Materials and Methods

### 2.1. Search Strategy

Randomized controlled trials (RCTs) were, respectively, retrieved by searching the following databases from the date of their inception to 30 April 2016: China National Knowledge Infrastructure (CNKI), Wanfang Database, China Science and Technology Journal Database (VIP), Chinese Biomedical Literature Database (CBM), PubMed, Cochrane Library, and EMBASE. In addition, CNKI was searched for conference articles and theses of Chinese Doctoral and Master. The search terms of Chinese databases were as follows: (“zhen ci” OR “zhen ci xue wei” OR “zhen ci liao fa” OR “dian zhen” OR “fu zhen” OR “tou zhen” OR “wen zhen jiu” OR “zhen jiu” OR “ji guang zhen”) AND (“jing xing xuan yun” OR “jing yuan xing xuan yun” OR “jin yuan xing tou yun” OR “zhui dong mai xing jing zhui bing” OR “zhui dong mai gong xue bu zu”). Search terms of English databases were as follows: (“manual acupuncture” OR “electro-acupuncture” OR “abdominal acupuncture” OR “scalp acupuncture” OR “warm-needle moxibustion” OR “acupuncture and moxibustion” OR “laser acupuncture”) AND (“cervical vertigo” OR “dizziness” OR “cervical spondylopasis of the vertebroarterial type” OR “vertebroarterial type”). At last, the reference lists of the selected studies and relevant reviews not found in the electronic searches were also manually searched to identify other appropriate studies.

### 2.2. Inclusion and Exclusion Criteria

Inclusion criteria were as follows.

#### 2.2.1. Types of Studies

The chosen trials were randomized controlled trials (RCTs), regardless of blinding, and were written in Chinese or English.

#### 2.2.2. Types of Participants

The studies included patients with CV who were diagnosed with the Diagnosis and Therapeutic Effects Criteria of TCM issued by the State Administration of TCM in 1994 [21] or diagnostic criteria proposed by the Second National Symposium on Cervical Spondylosis held in Qingdao in 1992 [22] or any other criteria deemed reasonable regardless of age, sex, or race.

#### 2.2.3. Types of Interventions and Control

The experiment group received acupuncture including manual acupuncture, electroacupuncture, abdominal acupuncture, scalp acupuncture, warm needle moxibustion, laser acupuncture, acupuncture, and moxibustion. RCTs that had control groups with no treatment, sham acupuncture, placebo control, drug therapy, and exercise therapy (such as cervical spondylosis training) were included. In addition, RCTs involving acupuncture combined with another therapy were also included if other therapies were equally used in both experimental and control groups.

#### 2.2.4. Types of Outcome Measures

The primary outcome was effectiveness. Therapeutic effects criteria referred to the Guiding Principles for Clinical Researches on New Chinese Drugs or TCM Effective Criteria in 1994. As the contents of the two standards are very similar, the effectiveness was presented by consistently using the following formula: rate (effectiveness) = *N*1 + *N*2 + *N*3/*N*, where *N*1, *N*2, and *N*3 are the number of patients cured, markedly improved, and improved and *N* is the sample size. Criteria for improvement after treatment are the following: cured: vertigo and all the accompanying symptoms disappeared, with a normal life and working ability; markedly improved: vertigo and the accompanying symptoms were much relieved; improved: vertigo was improved, but a slight spinning sensation still remained. Secondary outcomes were improvement rate of clinical symptoms including vertigo and headache (calculation formula and improvement criteria are similar to effectiveness) and the average blood flow velocity of vertebral-basilar artery and adverse reactions or adverse events.

Studies that met the following criteria were excluded: (1) trials that were duplicated studies, case reports, reviews, qualitative studies, or animal experiments were excluded; (2) trials that examined differences in various types of acupuncture methods or acupoints selection were excluded. Trials that designed a control of acupuncture plus moxibustion compared with moxibustion were also excluded; (3) trials that included participants with vertigo and dizziness but not caused by cervical spondylosis were excluded.

### 2.3. Data Extraction

For the included studies, two reviewers (Zhuanzhuan Hou and Shibing Xu) independently extracted the articles according to the established inclusion and exclusion criteria and read the full text for further determination. The collection of information included the author(s), publication year, study design, sample size, patients' characteristics, diagnostic criteria, acupuncture treatment process, details of the control, outcome measures (effectiveness, improvement rate of clinical symptoms, and change for average blood flow velocity of vertebral-basilar artery), withdrawal, and adverse events. The authors were contacted by e-mail for additional information if the data was unavailable. We used the intention-to-treat (ITT) analysis for dichotomous outcomes if possible.

### 2.4. Risk of Bias and Quality Assessment

Two reviewers independently evaluated the bias risk to included studies using the Cochrane Handbook, Version 5.1.0 [23]. The bias risk assessment tool involved seven aspects: random sequence generation (selection bias), allocation concealment (selection bias), binding of participants and personnel (performance bias), binding of outcome assessment (detection bias), incomplete outcome data (attrition bias), selective reporting (reporting bias), and other potential sources of bias. Three levels were used to evaluate the trials: low risk of bias (all the items were in low risk of bias), high risk of bias (at least one item was in high risk of bias), and unclear risk of bias (at least one item was in unclear risk of bias). This was independently evaluated by two reviewers (Zhuanzhuan Hou and Shibing Xu). Disagreements were resolved through arbitration from the third party (Huade Chen).

### 2.5. Statistical Analysis

*Cochrane.* Collaboration Review Manager software (RevMan 5.3) was used for data analyses. Relative risk (RR) was used for dichotomous data and mean difference (MD) was used for continuous variables. Outcomes were expressed with 95% confidence interval (CI), and *P* < 0.05 was considered statistically different between experimental and control groups. Before the data synthesis and analysis, heterogeneity test was done with the chi-squared test and the Higgins *I*^2^ test. *I*^2^ values of 25, 50, and 75% were nominally assigned as low, moderate, and high estimates, respectively [24]. A fixed-effects model was used when there was no significant heterogeneity (*I*^2^ < 50%) of the results of the studies. Otherwise, the random-effects model was used (*I*^2^ ≥ 50%). If the number of included trials was sufficient, subgroup analysis would be performed according to types of interventions, treatment process, and details of the control. A funnel plot would be carried out to assess asymmetry for publication bias, indicating the possibility of a small indistinct study bias [25]. More sensitivity analysis would be conducted to test the impact of the quality of included trials to the robustness of the meta-analysis results.

### 2.6. Level of Evidence

The Grading of Recommendations, Assessment, Development, and Evaluation (GRADE) was used to assess the level of evidence and summarize each outcome. GRADE is a method of grading the level of evidence developed by the GRADE Working Group [26, 27]. GRADE pro software (version 3.6 for Windows, Grade Working Group) was used.

## 3. Results

### 3.1. Study Selection

**Figure 1:**
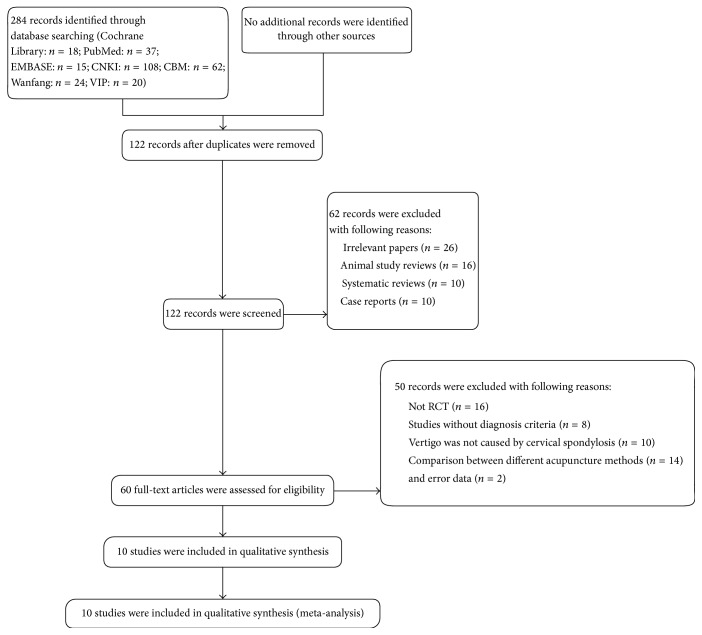
Flow chart of report selection process.

In this review, 284 articles (214 records from Chinese databases and 70 records from English databases) were retrieved from the databases listed above. After removing duplicates, 122 records remained. A total of 62 trials were excluded through reading the titles and abstracts due to lack of relevance. The full text of the remaining 60 articles was read and analyzed in detail, with 10 papers including an academic paper finally included for the systematic review. The process is shown in Figure 1.

### 3.2. Study Characteristics

The basic characteristics and main outcomes of the 10 trials were summarized in Tables 1 and 2. All of the included trials originated in China, with a total of 914 participants (467 in experiment groups and 447 in control groups). All included studies demonstrated no significant difference at baseline in gender, age, and disease duration. Diagnostic standard of five studies [28–32] was assessed by the Diagnosis and Therapeutic Effects Criteria of TCM issued by the State Administration of TCM in 1994. Five trials [33–37] adopted diagnostic standard which was diagnostic criteria proposed by the Second National Symposium on Cervical Spondylosis held in Qingdao in 1992. The interventions for the experiment groups included manual acupuncture (MA) in seven studies [28, 30, 31, 33, 35–37], electroacupuncture (EA) in two studies [29, 34], warm needle moxibustion in one study [32], and acupuncture combined with medication in two studies [29, 30]. The main acupoints selected were Baihui (DU20), Fengchi (GB20), Fengfu (GV16), and Tianzhu (BL10). The mean treatment time was approximately 2 to 4 weeks and treatment frequency was once a day. In the control groups, western medicine was used in night studies [28, 29, 31–37] and traditional Chinese medicine was adopted in one study [30]. The follow-up time was 1 month in one study [28] and 3 months in another study [34], and the rest did not mention the follow-up. Effectiveness was reported in all included studies [28–37], improvement rate of vertigo and headache in two studies [33, 36], increased average blood flow velocity of vertebral-basilar artery in three studies [32, 34, 35], and adverse reactions in three studies [28, 30, 32].

### 3.3. Quality of Included Studies

Through e-mail, we contacted the authors of studies [28, 32–34, 37] and determined random sequence generation ways of studies [28, 32–34, 37].

**Figure 2:**
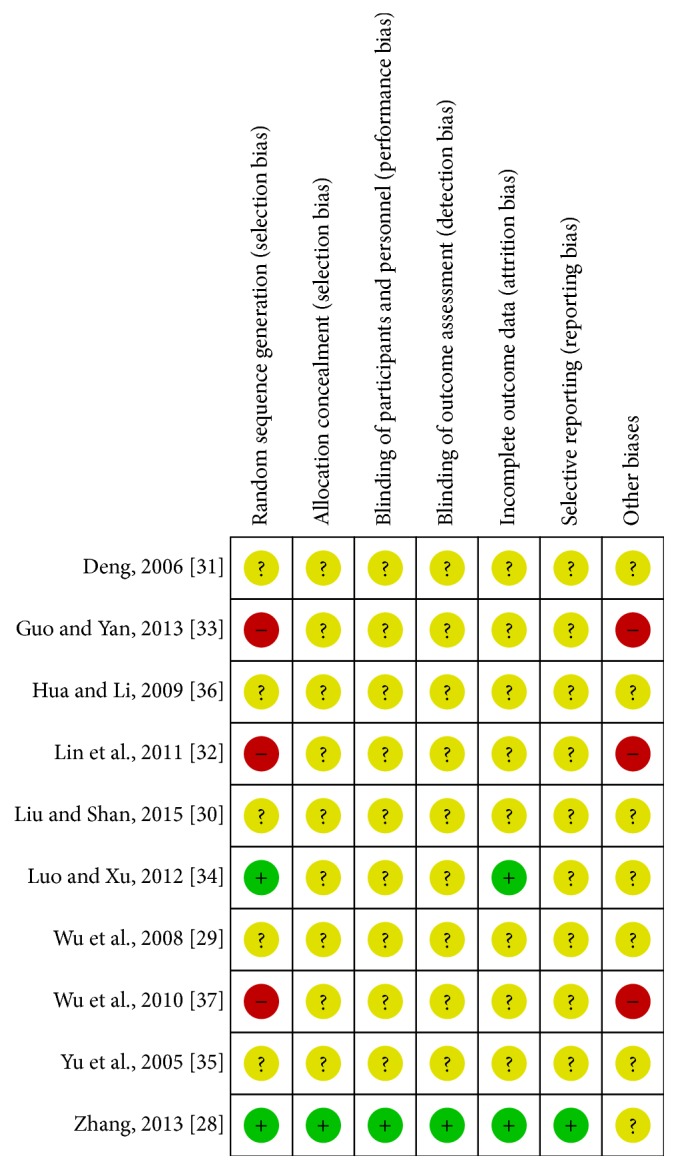
Summary of risk of bias of all included studies.

The authors of studies [28] provided ways of allocation concealment, binding, incomplete outcome data, and selective reporting with us. The authors of other studies did not respond to our e-mail. In all the included randomization, only 2 studies [28, 34] were considered low risk because of the right random sequence generation from computer randomization methods [28] and random number table [34]; three studies [32, 33, 37] of them were high risk according to the sequence of the attending doctor, and the information in the rest was not enough to make a judgement. One trial [28] used sealed envelope for allocation concealment and proper blinding method for outcome evaluators. Two trials [28, 34] mentioned expulsion case because of refusing further therapy. One trial [28] described withdrawal case owing to adverse reactions. But there was no information about the principle used for dealing with the missing data [38]. There was also no information related to intention-to-treat (ITT) analysis. So, we just conducted completer analysis. No reports mentioned that the research was approved by ethics committee and was registered. Therefore, all included trials were evaluated as having high risk of bias. The specific bias analysis of each test is shown in Figure 2.

### 3.4. Effectiveness

**Figure 3:**
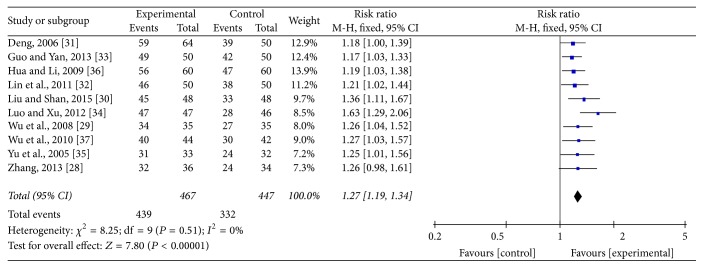
Forest of comparisons of total effectiveness between acupuncture group and medication group.

**Figure 4:**
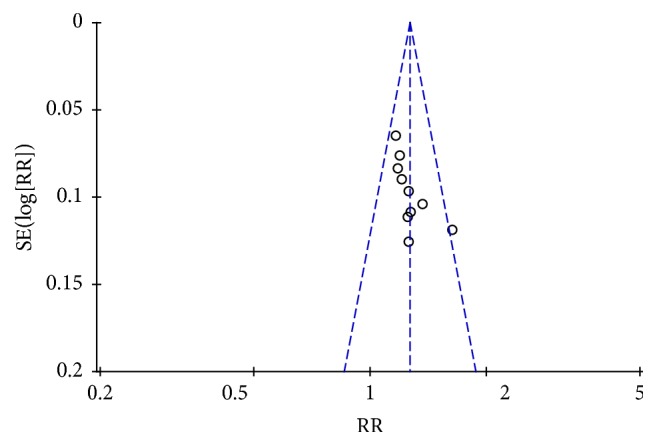
Funnel plot of the included trials in the effectiveness.

All included trials adopted effectiveness as the outcome by the following three main symptoms improvement levels: (1) clinical cured, (2) markedly improved, and (3) improved, a generally accepted rule in TCM which was performed in 1994. The total effective rate was considered as the percentage of the total of the sum of the three items. Pooled analysis of ten studies [28–37] with 467 patients in the acupuncture group and 447 in the medicine group revealed that acupuncture was significantly more effective than conventional medication (RR: 1.27; 95% CI: 1.19–1.34; *P* < 0.00001). As there was no homogeneity in the consistency of the trial results (*P* = 0.51; *I*^2^ = 0%), a fixed-effects model was applied (Figure 3). The subgroup analysis based on different acupuncture and drug categories found that the results did not change significantly (Table 3). Sensitivity analysis was performed to assess the stability of the results. When any single study was deleted, the corresponding pooled RR was changed slightly, with the statistically similar results indicating a good stability of the meta-analysis (Table 4). The graphic funnel plot of these ten studies appeared to be asymmetric, suggesting the possibility of publication bias (Figure 4).

### 3.5. Improvement Rate of Clinical Symptoms

**Figure 5:**
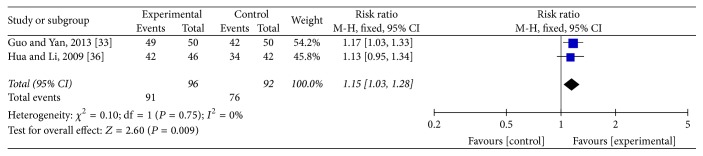
Forest of comparisons of improvement for vertigo: acupuncture versus medication.

**Figure 6:**
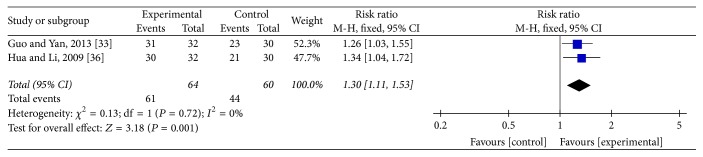
Forest of comparisons of improvement for headache: acupuncture versus medication.

Two studies [33, 36] reported the improvement rate of vertigo and headache. The meta-analysis showed that acupuncture was more effective than conventional medication in the improvement rate of vertigo (RR: 1.15; 95% CI: 1.03–1.28; *P* = 0.009) and headache (RR: 1.30; 95% CI: 1.1–1.53; *P* = 0.001). Heterogeneity test of vertigo (*P* = 0.75; *I*^2^ = 0%) and headache (*P* = 0.72, *I*^2^ = 0%) was homogenous and a fixed mode was used (Figures 5 and 6).

### 3.6. Vm of Vertebral-Basilar Artery

**Figure 7:**
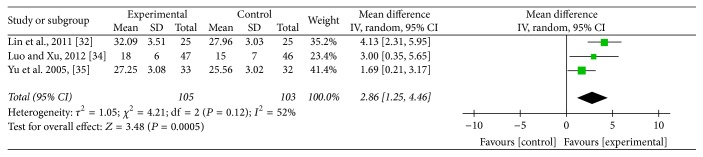
Forest of comparisons of improvement for Vm of left vertebral artery: acupuncture versus medication.

**Figure 8:**
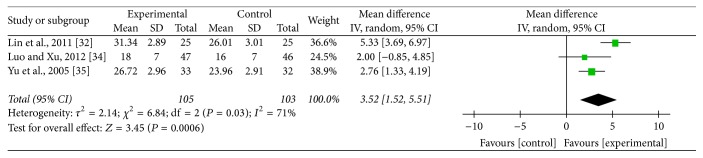
Forest of comparisons of improvement for Vm of right vertebral artery: acupuncture versus medication.

**Figure 9:**
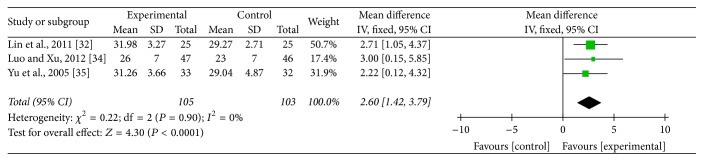
Forest of comparisons of improvement for Vm of basilar artery: acupuncture versus medication.

Three studies [32, 34, 35] adopted Vm of vertebral-basilar artery as an outcome which detected improvement of blood supply to the vertebral arteries. Meta-analysis revealed that acupuncture can increase more significantly Vm of left vertebral artery (MD: 2.86; 95% CI: 1.25–4.46; *P* = 0.0005), right vertebral artery (MD: 3.52; 95% CI: 1.52–5.51; *P* = 0.0006), and basilar artery (MD: 2.60; 95% CI: 1.42–3.79; *P* < 0.0001) compared with medication. As heterogeneity test for Vm of left vertebral artery (*P* = 0.12; *I*^2^ = 52%) and right vertebral artery (*P* = 0.03; *I*^2^ = 71%) was significant, a random mode was used (Figures 7 and 8). Meanwhile, heterogeneity test for Vm of basilar artery (*P* = 0.9; *I*^2^ = 0%) was homogenous and a fixed mode was adopted (Figure 9).

### 3.7. Adverse Effects

As shown in Table 1, only three trials [28, 30, 32] reported adverse reactions. In the study of Zhang [28], two cases with subcutaneous mild bruise and skin allergy reaction after needling occurred in the treatment group. Liu and Shan [30] and Lin et al. [32] reported no adverse effects in the acupuncture treatment group.

### 3.8. Level of Evidence

The levels of evidence as determined by GRADE ranged from very low to low (Table 5). Most of the studies did not report blinding, randomization sequence generation, or allocation concealment methods, so all outcomes were initially downgraded. In addition, the small number of participants of all outcomes also downgraded all outcomes except total effectiveness.

## 4. Discussion

### 4.1. Summary of Effectiveness

This systematic review and meta-analysis has shown that acupuncture was more effective than CMT in effectiveness, improvement rate of vertigo and headache, and improvement for average blood flow velocity of vertebral-basilar artery. In the subgroup analysis, the results did not change in different acupuncture methods and drug categories. Sensitivity analysis demonstrated that the results of this meta-analysis were stable. These seemingly positive results should be interpreted with caution mainly due to poor reporting, a limited number of studies, and the language limitation which meant that all chosen trials were written in Chinese or English in the inclusion criteria of this review.

### 4.2. Applicability of the Current Evidence

There were two systematic reviews [19, 20] that have been published during the last few years for assessing the effectiveness and safety of acupuncture for CV by evaluating effectiveness. Compared with previous systematic reviews, 8 papers [28–30, 32–34, 36, 37] of this review were not included in systematic reviews [19] and 18 papers of systematic reviews [19] had not been yet included in this review. In included criteria of systematic reviews [20], intervention type of experiment group was acupuncture plus massage therapy and intervention type of control group was acupuncture monotherapy or massage monotherapy. So, there is not a lot of correlation between this review and systematic reviews [20]. Compared with systematic reviews [19], in this review, we made rigorous control and excluded studies that examined differences in various types of acupuncture methods or acupoints selection. Importantly, we detect the average blood flow velocity of vertebral-basilar artery as an objective outcome to examine blood supply for the vertebral artery. Additionally, we conducted a subgroup analysis in different acupuncture methods and drug categories and sensitivity analysis which was performed by removing each study in sequence and recalculating the results. Meanwhile, compared with previous systematic reviews [19, 20], we did not have much progress in poor methodological quality, limited sample size of included studies, and drawing a definitive conclusion. Therefore, RCTs of further larger scale and high quality about acupuncture for CV should be first sought to provide more credible evidence.

Clinical heterogeneity may contribute to the difference in PICO (patients, intervention, control, and outcomes) of included studies. We conducted a subgroup analysis in different acupuncture methods and drug categories; however, it is difficult to assess this heterogeneity in terms of individual differences, all acupuncture details (needling depth, acupuncture manipulation, and needle retention time), drug categories, and various outcome measuring methods, as those detailed pieces of information are difficult to master and unify.

There are issues about high risk of bias happening in our analysis again. Its major responsibility was the lack of proper blinding and placebo effect such as sham acupuncture. The estimate of the intervention effect can be exaggerated when there is inadequate allocation concealment [39] or lack of blinding in trials where a subjective outcome is analyzed [40]. What made the blinding and sham acupuncture hard to be put into practice was the fact that acupuncture needed to be manipulated by a specialized doctor and blinding of the providers and patients owing to the nature of acupuncture would be hardly possible in clinical trials.

In the 10 included studies, participants ranged only from 65 to 120 in each trial (33 to 64 patients in the acupuncture group versus 32 to 60 patients in the control group). No trial reported a formal sample size calculation. Small number of sample size is likely to overestimate the acupuncture efficacy.

What is more, the primary outcome for this meta-analysis was effectiveness rate, which is a relatively subjective and positive measurement, and once effectiveness rate or improvement rate was used as a major measurement, it was likely to produce positive results and neglect most negative results in most acupuncture trials or RCTs of China. So it is worth noting that, with respect to efficacy rate, all meta-analysis results should be interpreted in caution, which will significantly affect the applicability of the current evidence provided by this systematic review.

### 4.3. Summary of Safety

In this review, only three [28, 30, 32] of 10 studies reported information about adverse reactions. Mild subcutaneous bruise and skin allergy reaction after needling occurred in one trial [28]. The two other trials [30, 32] reported no adverse reactions in the acupuncture treatment group. The remaining 7 clinical trials did not report any adverse reactions. However, only two trials [28, 29] mentioned follow-up time, but relevant information concerning the follow-up was not concerned. The absence of information on adverse reactions does not mean that the intervention is safe [41]. So, we cannot assure the safety of acupuncture in patients with CV. The long-term safety of acupuncture for CV still remains uncertain.

### 4.4. Implications for Further Practice and Research

Further studies of higher quality, larger sample size, and longer-term follow-up are needed for a more accurate analysis. Further study design should take into account the following points: (1) the design should utilize strict randomization, allocation concealment, and blinding, as these are the core standards of a well-designed RCT [39, 42]; (2) inclusion, exclusion, and diagnosis criteria should be clearly defined; (3) no language limited studies are needed; (4) all effects (positive and negative effects) about acupuncture for CV should be reported; (5) appropriate sample size and long-term follow-up are required; (6) clinical trials to report adverse events with more explanations are required [43].

## 5. Conclusion

This systematic review and meta-analysis based on current evidence suggested that acupuncture may be more effective in effectiveness and improvement of clinical symptom and average blood flow velocity of vertebral-basilar artery compared with conventional medicine therapy for CV. However, the findings should be insufficient to make a firm conclusion due to a lack of studies with high methodological quality. Further rigorously designed studies and higher-quality trials with larger sample size are necessary to confirm the effectiveness and safety of acupuncture for CV.

## Figures and Tables

**Table 1:** The basic characteristics of included studies.

Study	Study design	Sample size	Age (years)	Disease duration	Diagnostic criteria	Treatment duration (d)	Follow-up	Adverse reaction/event	Outcome
Zhang, 2013 [28]	RCT	E: 36	E: 45.72 ± 10.11	E: 11.15 ± 9.34 (m)	Criteria (1994)	28	1 (m)	E: BA	ER; AE
		C: 34	C: 45.68 ± 9.33	C: 9.49 ± 9.86 (m)				C: GR	

Wu et al., 2008 [29]	RCT	E: 35	E: 32–72	E: mean = 5.1 (a)	Criteria (1994)	20	2 (m)	NA	ER
		C: 35	C: 28–71	C: mean = 4.8 (a)					

Guo and Yan, 2013 [33]	RCT	E: 50	E: 53.68 ± 5.14	E: 21.90 ± 13.91 (m)	Criteria (1992)	20	NA	NA	ER; CSR
		C: 50	C: 52.42 ± 8.96	C: 19.24 ± 13.44 (m)					

Luo and Xu, 2012 [34]	RCT	E: 47	E: 55 ± 4	E: 12.0 ± 2.6 (m)	Criteria (1992)	20	NA	NA	ER; Vm
		C: 46	C: 58 ± 4	C: 10.1 ± 2.5 (m)					

Yu et al., 2005 [35]	RCT	E: 33	E: 46.3 ± 4.5	E: 2.6 ± 1.3 (a)	Criteria (1992)	14	NA	NA	ER; Vm
		C: 32	C: 47.1 ± 5.5	C: 4.5 ± 1.7 (a)					

Liu and Shan, 2015 [30]	RCT	E: 48	E: 48.42 ± 5.31	E: 5.64 ± 1.17 (a)	Criteria (1994)	10	NA	E: NO	ER; AE
		C: 48	C: 46.35 ± 10.21	C: 4.98 ± 1.24 (a)				C: NO	

Deng, 2006 [31]	RCT	E: 64	E: 51.5 ± 6.1	E: 38.2 ± 5.6 (d)	Criteria (1994)	30	NA	NA	ER
		C: 50	C: 48.1 ± 6.5	C: 36.2 ± 7.1 (d)					

Hua and Li, 2009 [36]	RCT	E: 60	E: 20–61	E: 1–10 (a)	Criteria (1992)	10	NA	NA	ER; CSR
		C: 60	C: 20–61	C: 1–10 (a)					

Wu et al., 2010 [37]	RCT	E: 44	E: 58 ± 13	E: 3.81 ± 0.59 (a)	Criteria (1992)	15	NA	NA	ER
		C: 42	C: 62 ± 13	C: 3.54 ± 0.55 (a)					

Lin et al., 2011 [32]	RCT	E: 50	E: 56.64 ± 9.87	E: 2.43 ± 2.82 (a)	Criteria (1994)	28	NA	E: NO	ER; Vm
		C: 50	C: 57.16 ± 9.42	C: 2.46 ± 2.39 (a)				C: GR	AE

E: experiment group; C: control group; a: annual; m: month; d: day; Criteria (1994): Diagnosis and Therapeutic Criteria of TCM (1994); Criteria (1992): Diagnostic criteria proposed by the Second National Symposium on Cervical Spondylosis (1992); NA: not available; BA: bruising and allergy of skin; GR: gastrointestinal reaction; ER: effective rate; AE: adverse event; CSR: improvement rate of clinical symptoms; Vm: average blood flow velocity of vertebral-basilar artery; TCM: traditional Chinese medicine.

**Table 2:** Details of acupuncture treatment and control interventions of included studies.

Study	Acupuncture rationale	Main acupoints	Details of needling					Control interventions
			Insertion depths	Responses elicited	Needle stimulation	Needle type	Retention time	
Zhang, 2013 [28]	TCM	PC6, GB34, GB20	10–20 mm	Deqi	MA	40 mm	30 min	Ligustrazine phosphate tablets (100 mg tid)

Wu et al., 2008 [29]	TCM	GB20, GV16, GV15	1 inch	Deqi	EA	0.35 mm *∗* 50 mm	30 min	Chuanxiongqin injection (120 mg + 5% GS 250 ml) Flunarizine (5 mg qn)

Guo and Yan, 2013 [33]	TCM	GV20, GV16, GB39 GB20, BL10, EX-B2	0.5–1 inch for GV20, GV6, EX-B2 0.5–0.8 inches for GB39, BL10, GB20	Deqi	MA	0.30 mm *∗* 40 mm	30 min	Flunarizine (5 mg qn) Nimesulide (0.1 g bid) Eperisone (50 mg tid)

Luo and Xu, 2012 [34]	TCM	GV20, GB20, GV16, EX-B2	NA	Deqi	EA	40 mm	30 min	Flunarizine (5 mg qn) Betahistine Mesilate tablets (6 mg tid)

Yu et al., 2005 [35]	TCM	am: CV12, LI11, LI4, ST36, SP9, ST40, SP6, LR3 SP10 pm: GB20, EX-B2	NA	NA	MA	NA	30 min	Betahistine hydrochloride injection (30 mg + 5% GS 250 ml)

Liu and Shan, 2015 [30]	TCM	GV20, EX-HN1, GB20, EX-B2	1.5 inches for GV20, EX-B2 1 inch for EX-HN1 0.8 inches for GB20	Deqi	MA	0.3 mm *∗* 50 mm	30 min	Banxiabaizhutianma decoction (1 dose qd)

Deng, 2006 [31]	TCM	GB19, GB20, BL9, BL10, TE23, GB8, GB4, GB5	1.5 inches–2 inches	NA	MA	NA	30 min	Betahistine hydrochloride 250 ml Flunarizine (5 mg qd)

Hua and Li, 2009 [36]	TCM	GB20, GB12, BL10, SI15 LI4, ST8, GV20	NA	Deqi	MA	0.3 mm *∗* 40 mm	30 min	Chuanxiongqin injection (80 mg + 5% GS 250 ml)

Wu et al., 2010 [37]	TCM	GB20, Gongxue, Jingpangsanzhen	1.5 inches–2 inches	NA	MA	NA	30 min	Nimodipine tablets (20 mg tid)

Lin et al., 2011 [32]	TCM	GB20, BL10, GV14 GB21, GV20, SI3, LU7	NA	Deqi	WNM	0.35 mm *∗* 50 mm	NA	Betahistine tablets (6 mg tid)

MA: manual acupuncture; EA: electroacupuncture; WNM: warm needle moxibustion; NA: not available.

Deqi: a sort of acid bilge feeling in patients and a sense in doctors which was vividly described as holding a float bobbing up and down when a fish was biting hook.

**Table 3:** The results of subgroup meta-analysis for total effectiveness.

Subgroup	Eligible Studies (number)	Acupuncture group (number)	Medication group (number)	RR/MD (95% CI)	*P* value	Heterogeneity test	Effect model
Acupuncture method							
Manual acupuncture	7 [28, 30, 31, 33–37]	355	316	1.23 (1.15, 1.32)	*P* < 0.00001	*I* ^2^ = 0%	Fixed
Electroacupuncture	2 [29, 34]	82	81	1.42 (1.09, 1.85)	*P* = 0.009	*I* ^2^ = 68%	Random
Warm needle moxibustion	1 [32]	50	50	1.21 (1.02, 1.44)	*P* = 0.03	—	—

Intervention type for treatment group							
Acupuncture	8 [28, 31–37]	384	364	1.25 (1.18, 1.34)	*P* < 0.00001	*I* ^2^ = 4%	Fixed
Acupuncture + drug	2 [29, 30]	83	83	1.32 (1.14, 1.52)	*P* = 0.0001	*I* ^2^ = 0%	Fixed

Drug categories of control group							
Ligustrazine	2 [28, 36]	124	110	1.19 (1.06, 1.33)	*P* = 0.02	*I* ^2^ = 0%	Fixed
Betahistine	2 [32, 35]	83	82	1.23 (1.07, 1.41)	*P* = 0.003	*I* ^2^ = 0%	Fixed
Ligustrazine + flunarizine	1 [29]	35	35	1.26 (1.04, 1.52)	*P* = 0.02	—	—
Betahistine + flunarizine	2 [31, 34]	111	96	1.37 (0.99, 1.90)	*P* = 0.05	*I* ^2^ = 81%	Random
Nimesulide + eperisone + flunarizine	1 [33]	50	50	1.17 (1.03, 1.33)	*P* = 0.02	—	—
Nimodipine	1 [30]	48	48	1.36 (1.11, 1.67)	*P* = 0.003	—	—
Traditional Chinese medicine	1 [37]	35	35	1.26 (1.04, 1.52)	*P* = 0.02	—	—

RR: risk ratio; MD: mean difference; 95% CI: 95% confidence interval.

**Table 4:** The results of the included studies through sensitivity analysis.

Excluded studies	Acupuncture group (number)	Medication group (number)	RR (95% CI)	*P* value	Heterogeneity test	Effect model
Zhang, 2013 [28]	431	413	1.27 (1.19, 1.35)	*P* < 0.00001	*I* ^2^ = 3%	Fixed
Wu et al., 2008 [29]	432	412	1.27 (1.19, 1.35)	*P* < 0.00001	*I* ^2^ = 3%	Fixed
Guo and Yan, 2013 [33]	417	397	1.28 (1.20, 1.37)	*P* < 0.00001	*I* ^2^ = 0%	Fixed
Luo and Xu, 2012 [34]	420	401	1.23 (1.16, 1.31)	*P* < 0.00001	*I* ^2^ = 0%	Fixed
Yu et al., 2005 [35]	434	415	1.27 (1.19, 1.35)	*P* < 0.00001	*I* ^2^ = 3%	Fixed
Liu and Shan, 2015 [30]	419	399	1.25 (1.18, 1.33)	*P* < 0.00001	*I* ^2^ = 0%	Fixed
Deng, 2006 [31]	403	397	1.28 (1.20, 1.36)	*P* < 0.00001	*I* ^2^ = 0%	Fixed
Hua and Li, 2009 [36]	407	387	1.28 (1.20, 1.36)	*P* < 0.00001	*I* ^2^ = 0%	Fixed
Wu et al., 2010 [37]	423	405	1.26 (1.19, 1.35)	*P* < 0.00001	*I* ^2^ = 3%	Fixed
Lin et al., 2011 [32]	417	397	1.27 (1.19, 1.35)	*P* < 0.00001	*I* ^2^ = 3%	Fixed

RR: risk ratio; 95% CI: 95% confidence interval.

**Table 5:** Level of evidence (GRADE).

Outcome	Effect		Number of participants (studies)	Quality of the evidence (GRADE)
	Relative effect (95% CI)	Absolute effect (95% CI)		
Total effectiveness	RR 1.27 (1.19 to 1.34)	201 more per 100 (from 141 more to 253 more)	914 (10 studies)	⊕⊕OO Low^1,4^

Vertigo	RR 1.15 (1.03 to 1.28)	124 more per 1000 (from 25 more to 231 more)	188 (2 studies)	⊕OOO Very low^1,2,4^

Headache	RR 1.3 (1.11 to 1.53)	220 more per 1000 (from 81 more to 389 more)	124 (2 studies)	⊕OOO Very low^1,2,4^

Left vertebral artery		MD 2.86 higher (1.25 to 4.46 higher)	208 (3 studies)	⊕OOO Very low^1,2,3,4^

Right vertebral artery		MD 3.52 higher (1.52 to 5.51 higher)	208 (3 studies)	⊕OOO Very low^1,2,3,4^

Basilar artery		MD 2.6 higher (1.42 to 3.79 higher)	208 (3 studies)	⊕OOO Very low^1,2,4^

^1^Most of them did not mention randomization process, allocation concealment, and blinding.

^2^Published evidence is limited due to a small number of trials, all of which are showing benefits.

^3^The heterogeneity is significant.

^4^Publication bias exists.
